# Oral microbiome as a co-mediator of halitosis and periodontitis: a narrative review

**DOI:** 10.3389/froh.2023.1229145

**Published:** 2023-08-31

**Authors:** Yeon-Hee Lee, Ji-Youn Hong

**Affiliations:** ^1^ Department of Orofacial Pain and Oral Medicine, Kyung Hee University School of Dentistry, Kyung Hee University Medical Center, Seoul, Republic of Korea; ^2^ Department of Periodontology, Periodontal-Implant Clinical Research Institute, School of Dentistry, Kyung Hee University, Seoul, Republic of Korea

**Keywords:** halitosis, oral malodor, periodontitis, oral microbiome, volatile sulfur compounds, bacteria

## Abstract

**Objective:**

Halitosis or oral malodor is an unpleasant odor from the oral cavity. However, although patients with periodontitis often complain of halitosis, their relationship has not been fully elucidated. We reviewed previous literature based on the hypothesis that the relationship between halitosis and periodontitis is mediated by the oral microbiome.

**Materials and methods:**

This narrative review sought to provide insight into the causative role of the oral microbiome in influencing halitosis and periodontitis. In addition, we tried to deepen knowledge related to the relationship between halitosis and periodontitis generated by the oral microbiome accumulated over the past 40 years.

**Results:**

From 1984 to 2023, a total of 106 papers that carefully and scientifically dealt with halitosis and periodontitis were included in this narrative review. Based on previous results, halitosis and periodontitis were closely related. For decades, researchers have taken an intriguing approach to the question of whether there is a relationship between halitosis and periodontitis. Central factors in the relationship between halitosis and periodontitis include volatile sulfur compounds (VSCs), the oral microbiota that produce VSCs, and the inflammatory response.

**Conclusions:**

Taken together, the more severe periodontitis, the higher the level of VSC in halitosis, which may be mediated by oral microbiome. However, the relationship between the occurrence, maintenance, and exacerbation of periodontitis and halitosis is not a necessary and sufficient condition for each other because they are complex interplay even in one individual.

## 1. Introduction

Halitosis or oral malodor is an unpleasant odor from the mouth and has various intraoral and extraoral causes. Patients with periodontitis often complain of halitosis, but the relationship between halitosis and periodontitis has not been fully elucidated. We reviewed previous literature based on the hypothesis that the relationship between halitosis and periodontitis is mediated by the oral microbiome. A narrative review was performed based on a search of PubMed and Google Scholar databases for articles on the role of the oral microbiome as an intermediary in halitosis and periodontitis. Keywords used in the search to find related articles are: “microbiome”, “microbiota”, “oral”, “oral cavity”, “saliva”, “halitosis”, “oral malodor”, “bad breath”, “volatile sulfur compounds”, “periodontitis”, “periodontal disease”, “bacteria”, “virus”, and “fungi”. Papers published in English in the last 40 years between January 1984 and March 2023 were filtered in this search. A total of 1,601 articles were retrieved from the PubMed and Google Scholar databases. Original researches and review articles that were related to our hypothesis and could directly or indirectly verify the hypothesis were selected. Articles were included allowing access to the abstract and full-text as well as the title of the article. Among the articles with the accessibility of full text, non-English articles were excluded. Conference papers, and working papers, web resources and bibliographic databases were also excluded. Finally, 106 articles were selected. The authors reviewed repeatedly over a 2-week period and mainly tried to verify the content and study design to determine whether the paper was suitable for this study.

## 2. Halitosis

### 2.1. Definition and prevalence of halitosis

Human breath is made up of very complex substances with various odors that can cause unpleasant situations such as halitosis. Halitosis is a word derived from the Latin words *halitus* (breathable air) and *osis* (pathological change), and refers to an unpleasant odor from the air and breath that originates from the mouth (1). Because halitosis affects conversations with others and furthermore, human relationships, it has caused many personal and social problems for millennia in worldwide. This undesirable halitosis is very common in both men and women and people of all ages (2). In meta-regression analysis, the combined prevalence of halitosis was 31.8% (3). In children, the prevalence of halitosis in the literature has been reported to range from 5% to 75% (4). The male:female ratio of halitosis prevalence ranges from 1.1:1 to 1.27:1, and halitosis is slightly more prevalent in males than in females (5, 6). Although the prevalence of halitosis varies depending on the diagnostic criteria or research method, there is no doubt that the prevalence is high, and understanding and exploration of halitosis should be continued.

### 2.2. Etiology of halitosis

The etiology of halitosis is multifactorial and the source is largely divided into oral and non-oral origin. Intra-oral origin of halitosis account for 80%–90% of all cases (4). First of all, tongue coating has been considered as a major factor in halitosis. The tongue coating contains food debris, desquamated epithelial cells, and blood cells, creating a perfect environment to nourish bacteria that produce volatile sulfur compounds (VSC) (7). Hydrogen sulfide and methyl mercaptan account for approximately 90% of VSCs (8). Self-cleaning of the tongue is difficult because of the complex anatomy of the tongue, especially at the posterior part of the tongue. The rough dorsal surface of the tongue with many papillae and deep fissures is easy for bacteria to adhere to, and it is difficult for saliva to self-purify (9, 10). Over time, the anaerobic environment of the tongue may change to facilitate colonization of Gram negative anaerobes (11). Conversely, a decrease in tongue coating thickness was associated with a decrease in the amount of *Porphyromonas gingivalis* and *Fusobacterium nucleatum* (12). With tongue cleaning with tongue scraper, a 75% reduction in VSCs was achieved (13).

The oral cavity contains multiple habitats, and the microbiome is diversely distributed in the tongue coating, saliva, teeth, buccal mucosa, soft and hard palate, gingival sulcus, tonsils, pharynx, and lips. There are 500–700 bacterial species found in the mouth, most of which can produce malodorous compounds that can cause bad breath (14, 15). In fact, the oral cavity is optimized for colonization and growth of microorganisms. The temperatures in the mouth can reach up to 37°C (changing between 34°C and 37°C). Humidity during exhalation can reach up to 96% (changing between 91% and 96%) in oral exhalation (16). These conditions can provide a suitable environment for bacterial colonization and growth.

Approximately 5%–9% of halitosis originates from non-oral structures such as the respiratory and gastrointestinal tract, and only 1% of bad breath is caused by medicine (Table 1) (17). Even among healthy people who do not have a history of halitosis and do not have periodontal disease, some people have halitosis because bacteria remain on the surface of the tongue (20). Oral bacteria break down organic substrates (such as glucose, mucins, peptides, proteins present in saliva, fissure fluid, oral soft tissue, and residual debris) and produce odorous compounds (18, 19). Halitosis is mainly formed by volatile organic compounds caused by pathological or non-pathological causes. These volatile organic compounds are sulfur compounds, aromatic compounds, nitrogen-containing compounds, amines, short-chain fatty acids, alcohols or phenyl compounds, aliphatic compounds and ketones (Table 2) (22).

**Table 1:** Oral and non-oral causes of halitosis (17–21).

Oral causes of halitosis	Non-oral causes of halitosis
Poor oral hygiene Xerostomia Tongue coating Gingivitis Periodontitis Peri-implantitis Acute necrotizing ulcerative gingivitis Adult and aggressive periodontitis Oral mucositis Oral ulceration Oral cancer Dental caries Candidiasis Fistula formation Pulp necrosis	Respiratory tract disease Sinusitis Tonsillitis Foreign body of the nose and/or lung Gastroesophageal reflux disease Infection Hematological disease Hepatic failure Leukemia Renal diseases Endocrine disease Diabetes Ketoacidosis Menstruation Metabolic syndrome Trimethylaminuria

**Table 2:** Volatile compounds that cause halitosis (22–24).

Family group	Compound name
Volatile sulfur compounds	Hydrogen sulfide Methyl mercaptan Dimethyl sulfide
Volatile aromatic compounds and amines	Urea Indole Skatole Pyridine Ammonia Putrescine Methylamine Dimethylamine Trimethylamine
Fatty acids or organic acids	Acetic acid Butyric acid Valeric acid Isovaleric acid Propionic acid
Alcohols	Ethanol Methanol Propanol
Volatile aliphatic compounds	Pentane Cyclobutane Cyclopropane
Aldehydes and ketones	Acetone Acetaldehyde Acetophenone Benzophenone

Among volatile compounds, VSCs are mainly responsible for bad breath from the oral cavity. Of course, organoleptic method performed by experts is considered the gold standard for diagnosing halitosis, but measurement of VSC levels using gas chromatography is objective and highly reliable (25, 26). Oral microbiome associated with halitosis, particularly gram-negative bacterial species and proteolytic anaerobes, inhabit and are active primarily in the tongue coating and periodontal pockets, and produce VSCs including hydrogen sulfide, methyl mercaptan, and dimethyl sulfide (23, 24). VSCs are produced by an enzymatic reaction by these bacteria of the sulfur-containing amino acids L-cysteine and L-methionine. Some bacteria also produce hydrogen sulfide and methyl mercaptan in serum (19, 27). Bacteria, the most active VSC producers, are presented in Table 3 (28).

**Table 3:** Bacteria that produce volatile sulfur compounds (28–33).

Volatile sulfur compounds	Bacteria
Hydrogen sulfide from cysteine	*Micros prevotii* *Baceroides spp*. *Centipedia periodontii* *Peptosteptococcus anaerobius*
Hydrogen sulfide from serum	*Prevotella intermedia* *Provotella loescheii* *Treponema denticola* *Porphyromonas gingivalis* *Selenomonas artermidis*
Methyl mercaptan from methionine	*Bacteroides spp*. *Eubacterium spp*. *Fusobacterium nucelatum* *Fusobacterium periodonticum*
Methyl mercaptan from serum	*Treponema denticola* *Porphyromonas gingivalis* *Porphyromonas endodontalis*

Wearing face masks during the Coronavirus Disease 2019 pandemic has increased concerns about the occurrence of halitosis (33, 34). Poor oral hygiene can be a key factor in the growth of the bacteria responsible for VSC production (17, 35). Oral-derived anaerobes can proliferate in chambers confined by face masks. Although the proliferation of microorganisms was observed on the inner surface of the mask, the amount of microbiome and VSC level did not significantly increase as the mask wearing time increased (33, 36). Additional research is needed to scientifically investigate whether wearing a mask in turn increases microorganisms and VSC levels.

### 2.3. Oral microbiome and halitosis

Recent research results are accumulating the fact that oral mircobiota can be a biomarker that distinguishes pathological conditions such as halitosis and periodontitis from oral health conditions. The oral bacteria species most related to halitosis are *Actinomyces spp.*, *Bacteroides spp.*, *Dialister spp.*, *Eubacterium spp.*, *Fusobacterium spp.*, *Leptotrichia spp.*, *Peptostreptococcus spp.*, *Porphyromonas spp.*, *Prevotella spp.*, *Selenomonas spp.*, *Solobacterium spp.*, *Veillonella spp.*, *and Tannerella forsythia* (32, 37, 38). Increased hydrogen sulfide and methyl mercaptan levels were associated with oral microbiota including *Prevotella spp.*, *Peptostreptococcus spp.*, *Eubacterium nodatum* and *Alloprevotella spp* (38). VSCs, such as hydrogen sulfide and methyl mercaptan, are the main constituents of oral malodor and are produced as end products of proteolytic processes by oral microorganisms. The main pathway of protein degradation is by metabolism of sulfur-containing amino acids by Gram-negative anaerobic bacteria. Especially, the most active producers of hydrogen sulfide are Gram-negative anaerobic bacteria such as *Prophyromonas gingivalis*, *Treponema denticola*, and *Tannerella forsythia*, which are members of the red complex that are associated with deep periodontal pockets (39–43). However, Gram-positive bacteria also play a crucial role in halitosis, by cleaving sugar chains from glycoproteins to provide protein, ultimately resulting in promotion of the production of VSCs by gram-negative anaerobes (44).

Compared to studies on halitosis and the species level of specific microbes, there are fewer studies on the relationship between halitosis at the genus and phylum level. The most dominant genera amongst the oral cavity microorganisms are *Alloprevotella*, *Leptotrichia*, *Peptostreptococcus*, *Prevotella*, and *Stomatobaculum* (38). The basic oral microbiota consists of the following phyla: *Actinobacteria*, *Bacteroidetes*, *Firmicutes*, *Fusobacteria*, and *Proteobacteria* (32). In recent deep learning approach, the genera *Rothia*, *Streptococcus*, and *Granulicatella* were more abundant in the healthy controls, whereas *Porphyromonas*, *Peptostreptococcus*, and *Veillonella* were more abundant in individuals with halitosis (45). At the genus level of bacteria involved in oral VSC production, representative hydrogen sulfide producers were genus *Fusobacterium*, *Neisseria*, and *Porphyromonas,* and methyl mercaptan producers were genus *Atopobium*, *Megasphaera*, *Prevotella*, *Selenomonas*, and *Veillonella*, respectively (46).

In the oral cavity, the anatomical part most associated with halitosis is the tongue. The microbiome of tongue coating, the main cause of halitosis, is reported more consistently at the phylum level, but at the species level may vary depending on sampling method, race, region, and inclusion criteria (38, 47). In 16S rRNA gene sequencing and next generation sequencing, the colonizing microbiota covering the tongue at the phylum level in healthy individuals included: *Actinobacteria*, *Spirochaetes*, *Fusobacteria*, *Bacteroidetes*, *Firmicutes*, and *Proteobacteria* (48–50). Regarding nonbacterial members, halitosis is associated with tongue coating of the tongue dorsum, where *Candida* species is the most commonly observed microorganism (51). Fungi also has the potential to increase VSC levels, and methyl mercaptan concentration was related with the presence of Candida albicans (52). *Escherichia coli* (*E. coli*) is a candidate microorganism that causes halitosis by converting cysteine to ammonia using cysteine dehydratase and reducing nitrate to ammonia (53, 54). In addition, one of the major contributors to trimethylamine production is *E. coli* (55).

Given the relationship between viral infections and bad breath, bad breath emerged as a problem during the coronavirus disease 2019 (COVID-19) pandemic. Halitosis has been reported in cases actively infected with severe acute respiratory syndrome coronavirus-2 (SARS-CoV-2) (56). Even, some have had new-onset halitosis during infection with SARS-CoV-2 (57). In acutely infected patients with COVID-19, decreased salivary flow may cause xerostomia and thus more likely mediate the occurrence of halitosis (58). Halitosis was strongly associated with epithelial structural alterations with degeneration of the keratinized epithelium (59). Epithelial changes in the tongue dorsum may be caused by the highly expressed angiotensin converting enzyme 2 receptor, the SARS-CoV-2 binding site (60).

However, as there are many factors that influence the formation of the oral microbiota, including temperature, humidity, salivary volume, pH, oxygen level, and the rate of mucosal cell shedding (61), additional studies on microorganisms affecting halitosis are needed. According to a recent sex-stratified metagenome-genome-wide-association study, dental calculus, bleeding frequency of gums, and high-fat and high-sugar diet frequency were factors influencing oral microbiome compositions, and sex differences of saliva microbiome composition was observed (62). hormonal profiles were the main cause of sex differences in the phylum-level of the gut-microbiome (63). However, the distribution of the oral microbiome did not differ according to the hormonal cycle and menopause of healthy women (64). Further research is needed to determine whether there are sex-specific differences in the oral microbiome in halitosis patients.

In oral cancer, the changed profile of the oral microbiota is considered as a reservoir of a diagnostic and prognostic biomarkers (65). Additionally, the abundant genus *Porphyromonas* has been associated with the development of oral and digestive tract cancers, as well as halitosis and periodontitis (66, 67). A peculiarly pungent halitosis was confirmed in the breath of patients with oral malignancies (68). Malodorous agents responsible for carcinogenesis are hydrogen sulfide and acetaldehyde. Another VSC is dimethyl sulfide, which is primarily responsible for extraoral or bloodborne halitosis, but can also contribute to oral malodor (21). Ketones, such as acetone, benzophenone, and acetophenone, are present both in the alveoli of the lungs and in the mouth air. Indole and dimethyl selenide also cause bad breath. Halitosis creates social and psychological disadvantages for individuals, and these situations affect individual's relation with other people (16). To re-establish the symbiosis of the oral cavity in patients with halitosis and to develop new strategies to help substantially reduce VSCs, it is necessary to identify predictive microbiome biomarkers.

## 3. Periodontitis

### 3.1. Definition and prevalence of periodontitis

Periodontitis is a chronic inflammatory condition in the supporting tissues of tooth that causes progressive destruction of attachment apparatus including alveolar bone, periodontal ligament and cementum. It is manifested as clinical attachment loss, periodontal pocketing, gingival bleeding and radiographic alveolar bone loss (69). Characteristics of irreversible destruction in periodontal tissues might result in tooth loss when the progression of the disease was severed, which leads to the problems in esthetics, impaired oral function and quality of life.

The Global Burden of Disease 2015 study reported that oral diseases are highly prevalent involving 3.5 billion people worldwide with untreated dental caries, severe periodontitis, and severe tooth loss (70). Approximately, 10.8% of the global population showed severe periodontitis that was the sixth-most prevalent health problems. In addition, complete edentulism and severe tooth loss was reported to be 2.3% and 2.4%, respectively, which led to the global burdens of direct and indirect economic costs, and intangible costs associated with social activities (71).

### 3.2. Etiology of periodontitis

Periodontitis is a disease mediated by dental biofilm (dental plaque) that contains hundreds of species of bacteria and possibly viruses and fungi which are together called oral microbiome, and host's immune response to the plaque biofilm. In healthy gingiva, commensal microflora in oral microbiome exists in harmony with the host, hence called symbiosis (72). When the bacterial deposits accumulate at the gingival crevice with poor oral hygiene, changes of the plaque mass and microbial compositions result in the gingivitis initially that represents inflammation in the periodontal soft tissues but no loss of periodontal support. However, chronic subgingival microbial colonization with pathogenic compositional shift (dysbiosis) can cause destruction of periodontal supporting tissue either by hyporesponsive or hyper-responsive inflammatory reaction (73). Intrinsic host genetic factors and acquired environmental stressors can modulate immune response, which affect the individual susceptibility to the disease (74). In addition, periodontitis can be associated with other chronic non-communicable diseases such as diabetes and cardiovascular disease by sharing common inflammatory pathways (71).

In oral cavity, various habitats including tooth surface (supragingival and subgingival), crevicular epithelium, buccal mucosa, tongue, tonsil and palate have unique micro-environmental conditions to form niches for heterogenous oral microbiome. The oral microbiome comprises bacteria, archaea, fungi, protozoa and viruses, which exists as planktonic phase in saliva or attached to oral surfaces such as a plaque biofilm (72). Subgingival microbial colonization at the root surface is critical for the development of periodontal disease as it can evade shear forces and is under the microenvironmental conditions with low redox potential and gingival crevicular fluid for nutritional source that favors the growth and maintenance of various species including anaerobic pathogens (30).

### 3.3. Oral microbiome and periodontitis

More than 500 species of bacteria have been detected in the subgingival plaque and the current high-throughput molecular technologies are extending the information about highly diverse community in oral microbiome (31). In previous model of plaque development in healthy gingiva, gram-positive cocci and rods are dominant in subgingival microbiome. *Actinomyces* spp. and *Streptococcus* spp. are the representative species which act as early colonizers and form early dental plaque. The second most frequent species appears to be the gram-negative rod *Fusobacterium nucleatum* which is the second colonizer that bridges different kinds of bacteria during the plaque maturation. However, recent studies that integrated spectral imaging with high-throughput sequencing data reported a radially arranged, multigenus consortium in the microbiome of dental plaque (75–77). The spatial organization of bacterial consortium consisted of filamentous *Corynebacterium* as a primary framework foundation that favored growth of *Streptococcus* at the periphery in a “hedgehog” appearance. Other taxa identified as regular participants in periphery of hedgehog structure included *Porphyromonas*, *Haemophilus/Aggregatibacter*, *Neisseriaceae*, *Fusobacterium*, *Leptotrichia*, *Capnocytophaga*, and *Actinomyces*, which were localized in a well-defined zone according to the microenvironments they were engaged in (76).

Undisturbed plaque accumulation causes shifts in subgingival composition to increase gram-negative rods, filaments and spirochetes accompanied by the clinical gingival inflammation, which can be reversed to the healthy state after removal of the plaque. *Prevotella* spp., *Selenomonas* spp., and *Fusobacterium nucleatum* subspecies *polymorphum* are enriched with the development of gingivitis while relative abundance of gram-positive species such as *Rothia dentocariosa* decrease significantly (78). In addition, total bacterial biomass increases approximately 3-log which makes the influence of proportional changes much larger.

Dysbiosis associated with periodontitis can be characterized by profound compositional shifts to diverse groups of gram-negative species in subgingival microbiome which are even different from those found in gingivitis (79). Red complex triad comprising *Porphyromonas gingivalis*, *Treponema denticola*, and *Tannerella forsythia* described in the fundamental study by Socransky et al. is one of the enriched species and strongly associated with periodontal destruction (39). They possess virulence factors with high protease activity and act as keystone pathogens that orchestrate inflammatory process by involving with microbial shifts to diseased state, acquisition of nutrition from host and growth of pathobionts to increase host response (80). For example, *P. gingivalis* can trigger imbalance between bacteria and host response by avoiding host immune component Toll-like receptors and complements, which brings out changes in the relative abundance of other bacteria. Besides the increased diversity of periodontitis-associated microbiome, some species including *Campylobacter gracilis* and *Fusobacterium nucleatum* are identified consistent with the proportions in health and periodontitis which are classified as core species (79). It shows that the species associated with health state are still present and dysbiosis can be described again as shifts in the dominant species. In addition, total biomass increases significantly which aggravates the host interaction to higher microbial load for both core and periodontitis-associated species.

## 4. Oral microbiome, halitosis, and periodontitis

Clinically, oral malodor is very common in patients with periodontitis. For decades, researchers have taken an intriguing approach to presenting scientific evidence for the relationship between bad breath and periodontitis (81–83). However, the strength of scientific evidence for a link between halitosis and periodontitis is not strong. In one case-control study, in 81.7% of patients with periodontitis, halitosis was found as a comorbidity, which was significantly higher than in healthy controls (58.3%) (84). In on observational study, approximately 90% of halitosis causes were of oral origin, with tongue coating (43%) being the most common cause, followed by gingivitis or periodontitis (11%), and a combination of the two subtypes of periodontal diseases (18%) (83). However, it is controversial whether tongue coating is a direct cause of gingivitis or periodontitis. Tongue coating thickness was not significantly associated with gingivitis and bleeding on pocket probing (85).

In studies examining tongue-coating microbes, *fusobacterium periodonticum*, *neisseria mucosa*, and *aggregatibacter segnis* were associated with tongue coating (86). The Extended Human Oral Microbiome Database (eHOMD) was updated in 2017 and found that six major phyla including *Firmicutes*, *Actinobacteria*, *Proteobacteria*, *Fusobacteria*, *Bacteroidetes* and *Spirochetes*, constitute 96% of total oral bacteria (87). At the phylum level, the proportions of *Actinobacteria* and *Spirochaetes* were higher in the tongue coating than in other areas of the oral cavity (87). When the distribution of 7 phyla, 27 genera and 825 operational taxonomic units of dorsal tongue microbes in halitosis patients was compared with that of healthy controls, there was no difference at the phylum level, and there were significant differences in some genus and species including *Aggregatibacter*, *Campylobacter*, *Capnocytophaga*, *Prevotella*, and *Treponema* (88). In children, *Leptotrichia wadei*, *Peptostreptococcus stomatis* and *Prevotella shahii* were higher in the tongue coatings of children with halitosis than in children without halitosis (89). Microbiota related to tongue coating or oral hygiene should continue to develop microbial-based biomarkers for use in the diagnosis of periodontitis as well as halitosis.

**Figure 1:**
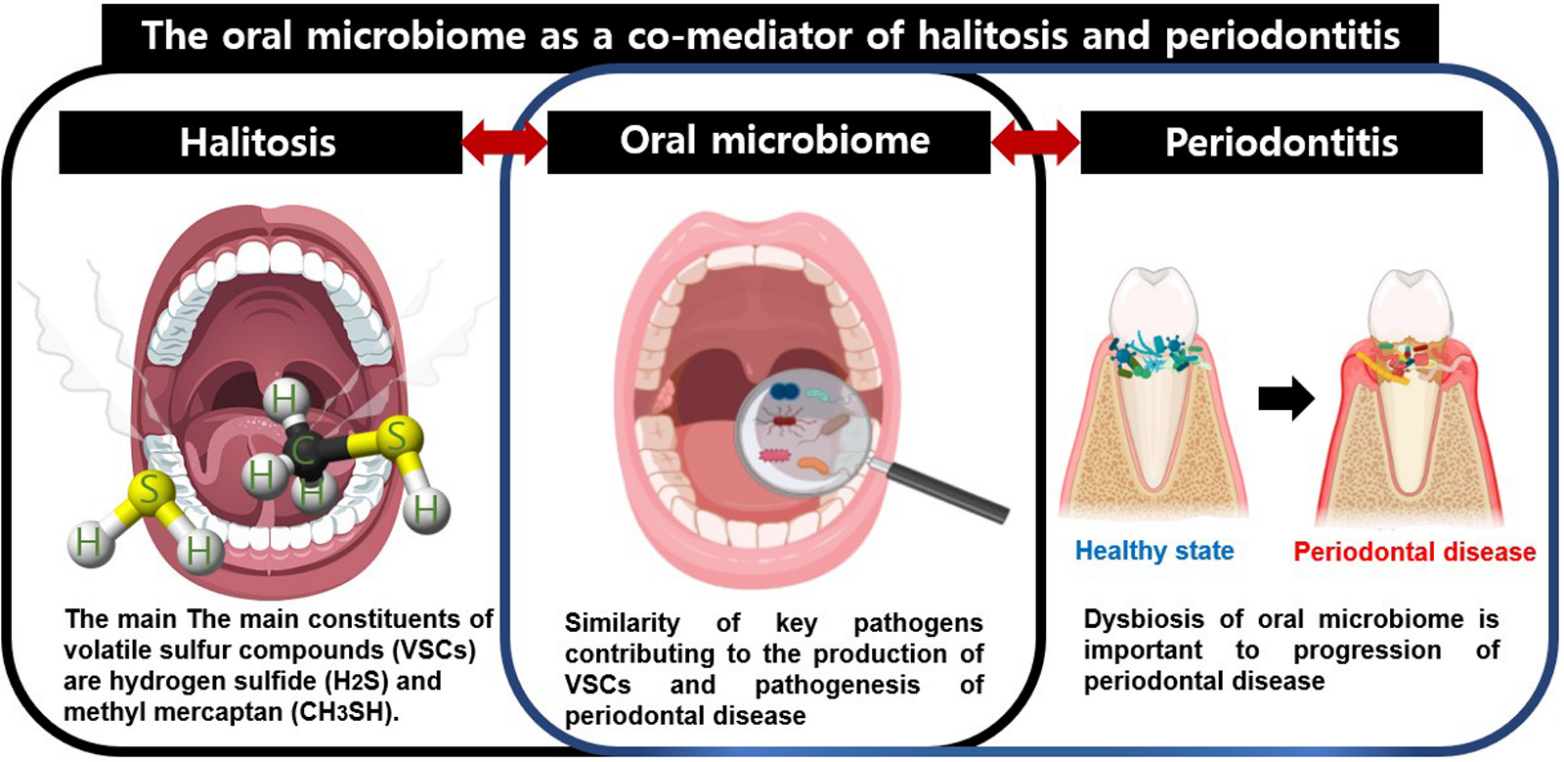
Oral microbiome linking halitosis and periodontitis.

Nevertheless, an open mind should be maintained about the possibility that microbes could be co-mediators of the halitosis and periodontitis. The term co-mediator used in this study refers to microorganisms that can be simultaneously involved in two diseases and can interplay between the two diseases. Since most of the microorganisms responsible for bad breath are involved in periodontitis, the oral microbiome may be a co-mediator of these two diseases. Central factors in the relationship between halitosis and periodontitis include VSCs, the oral microbiota that produce VSCs, and the inflammatory response. Accompanying inflamed periodontal pockets in periodontitis provide a stable habitat for the oral microbiota, which enhances hydrogen sulfide production (15). The tongue dorsum is also a major habitat for periodontal disease-associated bacteria such as *Porphyromonas gingivalis* and *Treponema denticola* (90). Among the various related oral microbiota, *Porphyromonas gingivalis*, *Treponema denticola*, and *Tannerella forsythia* are closely related to the progression and exacerbation of periodontitis, as well as to the occurrence of halitosis by increasing the production of VSCs (91–93). Thus, key pathogens in periodontal disease can produce VSCs and increase the incidence of halitosis, and oral dysbiosis is important in the development of halitosis, as well as in the progression of periodontal disease. Therefore, oral microorganisms can be considered as co-mediators of these two diseases (Figure 1). To the best of our knowledge, few studies have investigated the prevalence of periodontitis in patients with halitosis. Specifically, increased levels of VSCs in the oral cavity were associated with the number and extent of periodontal pockets deeper than 3 mm (94). Patients with one or more periodontal pockets greater than 5 mm in depth had a 30% increase in VSC values compared to normal subjects (95). Aggravation of the severity of periodontitis was associated with an increase in bad breath. Conversely, curettage and periodontal surgery can reduce VSC concentration (96). In a more recent study, *Fusobacterium nucleatum*, *Capnocytophaga gingivalis,* and *Campylobacter showaei* correlated with reduced VSC levels after periodontal therapy (97). When water-flossing and toothbrushing were performed together, the level of halitosis was lowered at week 12, and the dental plaque microbiota was changed. When water-flossing and toothbrushing were performed together, the level of halitosis was lowered at week 12, and compared to the case of toothbrushing alone, *Prevotella* at genus level and *Prevotella intermedia* at species level were significantly reduced among dental plaque microbiota (97). Gram-negative anaerobes, the main cause in both halitosis and periodontitis, mainly inhabit the posterior part of the tongue (98). It has even been suggested that the coating of tongue dorsum acts as a reservoir to periodontal pathogens (90). Therefore, tongue cleaning and maintaining good oral hygiene not only reduce gram-negative anaerobes, but also prevent nutrient supply to them, thereby reducing VSC production in healthy subjects as well as patients with periodontitis.

Two sulfide gases, hydrogen sulfide and methyl mercaptan, are the main causes of halitosis in VSCs, but methyl mercaptan seems to play a more important role in relation to periodontitis. First of all, the reason why methyl mercaptan has more adverse effects on patients with halitosis and periodontitis than hydrogen sulfide is that it has the permeability of oral mucosa (99). Particularly high concentrations of methyl mercaptan were found among VSCs in subjects with probing depths greater than 4 mm (82). Moreover, methyl mercaptan can dimerize to dimethyl sulfide, and since sulfide is considered highly cytotoxic, methyl mercaptan may accelerate periodontal disease progression (100). Tongue biofilm and plaque was the major source of higher amounts of VSC, especially methyl mercaptan, in patients with periodontitis (101, 102). Patients with periodontitis had higher VSC levels compared to healthy controls, and their greater subgingival bacterial diversity was positively associated with hydrogen sulfide levels (103). In the tongue biofilm metatranscriptome analysis, over-expression of genes related to cysteine degradation into hydrogen sulfide was observed in patients with halitosis (104). Based on previous results, it can be concluded that halitosis and periodontitis are closely related. Oral microorganisms involved in the occurrence and progression of halitosis and periodontitis can promote the production of VSCs, and subsequently, individuals affected by these diseases are affected biologically, chemically, and locally and systemically, and eventually halitosis and periodontitis may worsen. Conversely, by early detection of changes in oral microorganisms and controlling the interaction of these microorganisms, the two diseases can be effectively controlled simultaneously.

However, even within individuals, the interplay between halitosis and periodontitis is very complex, making it difficult to derive a single relationship. For example, even in one individual, the relationship between halitosis and periodontal diseases may be different according to the site of periodontitis and their local and/or systemic condition, and this relationship may change with the increase of age (105). Thus, it should be noted in the interpretation of the clinical results that halitosis may occur temporarily as part of the physiological phenomenon or mentally even in normal people, so the occurrence, maintenance, and exacerbation of the two diseases are not necessary and sufficient conditions for each other. Furthermore, halitosis is affected by several systemic diseases such as gastrointestinal disease, respiratory system problems, hepatic disease, renal disease, diabetes mellitus, and several metabolic diseases (17, 106). Therefore, when considering the oral microbiota in the relationship between halitosis and periodontitis, it is important to understand the diversity of the oral microbiota and how it fluctuates under disease/disturbing conditions. According to eHOMD results published in 2017, only 70% are culturable species, of which only 57% have been named (87). That is, much of the oral microbiome remains a mystery. To have clear conclusions, high-throughput epidemiologic investigations and analysis based on the advanced technology for oral microbiome are additionally required.

## 5. Conclusion

Taken together, specific oral microbiome profiles and dysbiosis may be associated with halitosis and periodontitis. The direct cause of bad breath is VSC produced by the oral microbiome. Changes in the oral microbiome and VSC production are also involved in the progression of periodontitis. In other words, specific microbiota can interplay and mediate the onset and progression of two diseases, halitosis and periodontitis. Although a clear relationship between halitosis and periodontitis is evident, it is unclear whether the oral microbiome mediates this relationship. Prospective, large-scale, clinical studies and animal experiments are needed to demonstrate the role of the oral microbiome in the relationship between halitosis and periodontitis. In the not-too-distant future, if biomarkers that co-mediate halitosis and periodontitis are clarified, which may have full potential to lead to early diagnosis and improve quality of life for patients with these diseases, and to lighten the burden on clinicians.

## References

[1] Hine MK. Halitosis. J Am Dent Assoc. (1957) 55:37–46. 10.14219/jada.archive.1957.0147 13438639

[2] Madhushankari GS Yamunadevi A Selvamani M Mohan Kumar KP Basandi PS. Halitosis—an overview: part-I—classification, etiology, and pathophysiology of halitosis. J Pharm Bioallied Sci. (2015) 7:S339–43. 10.4103/0975-7406.163441 26538874 PMC4606616

[3] Silva MF Leite FRM Ferreira LB Pola NM Scannapieco FA Demarco FF Estimated prevalence of halitosis: a systematic review and meta-regression analysis. Clin Oral Investig. (2018) 22:47–55. 10.1007/s00784-017-2164-5 28676903

[4] Motta LJ Bachiega JC Guedes CC Laranja LT Bussadori SK. Association between halitosis and mouth breathing in children. Clinics. (2011) 66:939–42. 10.1590/S1807-59322011000600003 21808855 PMC3129960

[5] Kim SY Sim S Kim SG Park B Choi HG. Prevalence and associated factors of subjective halitosis in Korean adolescents. PLoS One. (2015) 10:e0140214. 26461837 10.1371/journal.pone.0140214 PMC4603949

[6] Nazir MA Almas K Majeed MI. The prevalence of halitosis (oral malodor) and associated factors among dental students and interns, Lahore, Pakistan. Eur J Dent. (2017) 11:480–5. 10.4103/ejd.ejd_142_17 29279674 PMC5727733

[7] Kamaraj RD Bhushan KS Vandana KL. An evaluation of microbial profile in halitosis with tongue coating using PCR (polymerase chain reaction)- a clinical and microbiological study. J Clin Diagn Res. (2014) 8:263–7. 10.7860/JCDR/2014/6213.3856 PMC3939570 24596791

[8] Payne D Gordon JJ Nisbet S Karwal R Bosma ML. A randomised clinical trial to assess control of oral malodour by a novel dentifrice containing 0.1%w/w o-cymen-5-ol, 0.6%w/w zinc chloride. Int Dent J. (2011) 61:60–6. 10.1111/j.1875-595X.2011.00051.x 21762157 PMC9374965

[9] Emura S Tamada A Hayakawa D Chen H Yano R Shoumura S. Morphology of the dorsal lingual papillae in the blackbuck, Antilope cervicapra. Okajimas Folia Anat Jpn. (1999) 76:247–53. 10.2535/ofaj1936.76.5_247 10693328

[10] Sara B Giuseppe M Adelaide CM. Dorsal lingual surface and halitosis: a morphological point of view. Acta Stomatol Croat. (2016) 50:151–7. 10.15644/asc50/2/8 27789913 PMC5080560

[11] Deo PN Deshmukh R. Oral microbiome: unveiling the fundamentals. J Oral Maxillofac Pathol. (2019) 23:122–8. 31110428 10.4103/jomfp.JOMFP_304_18 PMC6503789

[12] Patil P Patil L Triveni MG Usha GV Shah R Kumar ABT. Efficacy of antimicrobial photodynamic therapy on the tongue surface in the management of halitosis—a real-time polymerase chain reaction analysis. Photodiagnosis Photodyn Ther. (2022) 39:102989. 10.1016/j.pdpdt.2022.102989 35792253

[13] Pedrazzi V Sato S De Mattos Mda G Lara EH Panzeri H. Tongue-cleaning methods: a comparative clinical trial employing a toothbrush and a tongue scraper. J Periodontol. (2004) 75:1009–12. 10.1902/jop.2004.75.7.1009 15341360

[14] Aas JA Paster BJ Stokes LN Olsen I Dewhirst FE. Defining the normal bacterial flora of the oral cavity. J Clin Microbiol. (2005) 43:5721–32. 10.1128/JCM.43.11.5721-5732.2005 16272510 PMC1287824

[15] Chattopadhyay I Verma M Panda M. Role of oral microbiome signatures in diagnosis and prognosis of oral cancer. Technol Cancer Res Treat. (2019) 18:1533033819867354. 10.1177/1533033819867354 31370775 PMC6676258

[16] Vali A Roohafza H Hassanzadeh Keshteli A Afghari P Javad Shirani M Afshar H Relationship between subjective halitosis and psychological factors. Int Dent J. (2015) 65:120–6. 10.1111/idj.12153 25753023 PMC9376520

[17] Aylıkcı BU Colak H. Halitosis: from diagnosis to management. J Nat Sci Biol Med. (2013) 4:14–23. 10.4103/0976-9668.107255 23633830 PMC3633265

[18] Mcnamara TF Alexander JF Lee M. The role of microorganisms in the production of oral malodor. Oral Surg Oral Med Oral Pathol. (1972) 34:41–8. 10.1016/0030-4220(72)90271-X 4504316

[19] Persson S Claesson R Carlsson J. The capacity of subgingival microbiotas to produce volatile sulfur compounds in human serum. Oral Microbiol Immunol. (1989) 4:169–72. 10.1111/j.1399-302X.1989.tb00246.x 2639302

[20] Wåler SM. On the transformation of sulfur-containing amino acids and peptides to volatile sulfur compounds (VSC) in the human mouth. Eur J Oral Sci. (1997) 105:534–7. 10.1111/j.1600-0722.1997.tb00241.x 9395120

[21] Harvey-Woodworth CN. Dimethylsulphidemia: the significance of dimethyl sulphide in extra-oral, blood borne halitosis. Br Dent J. (2013) 214:E20. 10.1038/sj.bdj.2013.329 23579164

[22] Thorn RM Greenman J. Microbial volatile compounds in health and disease conditions. J Breath Res. (2012) 6:024001. 10.1088/1752-7155/6/2/024001 22556190 PMC7106765

[23] Scully C Rosenberg M. Halitosis. Dent Update. (2003) 30:205–10. 10.12968/denu.2003.30.4.205 12830698

[24] Ferguson M Aydin M Mickel J. Halitosis and the tonsils: a review of management. Otolaryngol Head Neck Surg. (2014) 151:567–74. 10.1177/0194599814544881 25096359

[25] Van Den Velde S. Van Steenberghe D. Van Hee P., and Quirynen M. (2009). Detection of odorous compounds in breath. J Dent Res 88, 285–9. 10.1177/0022034508329741 19329466

[26] Falcão DP Miranda PC Almeida TFG Scalco M Fregni F Amorim RFB. Assessment of the accuracy of portable monitors for halitosis evaluation in subjects without malodor complaint. Are they reliable for clinical practice? J Appl Oral Sci. (2017) 25:559–65. 10.1590/1678-7757-2016-0305 29069154 PMC5804393

[27] Persson S Edlund MB Claesson R Carlsson J. The formation of hydrogen sulfide and methyl mercaptan by oral bacteria. Oral Microbiol Immunol. (1990) 5:195–201. 10.1111/j.1399-302X.1990.tb00645.x 2082242

[28] Krespi YP Shrime MG Kacker A. The relationship between oral malodor and volatile sulfur compound-producing bacteria. Otolaryngol Head Neck Surg. (2006) 135:671–6. 10.1016/j.otohns.2005.09.036 17071291

[29] Abusleme L Dupuy AK Dutzan N Silva N Burleson JA Strausbaugh LD The subgingival microbiome in health and periodontitis and its relationship with community biomass and inflammation. Isme j. (2013) 7:1016–25. 10.1038/ismej.2012.174 23303375 PMC3635234

[30] Mombelli A. Microbial colonization of the periodontal pocket and its significance for periodontal therapy. Periodontol 2000. (2018) 76:85–96. 10.1111/prd.12147 29193304

[31] Curtis MA Diaz PI Van Dyke TE. The role of the microbiota in periodontal disease. Periodontol 2000. (2020) 83:14–25. 10.1111/prd.12296 32385883

[32] Hampelska K Jaworska MM Babalska Z Karpiński TM. The role of oral Microbiota in intra-oral halitosis. J Clin Med. (2020) 9. 10.3390/jcm9082484 32748883 PMC7465478

[33] Lee Y-H Kim H Heo DW Ahn I-S Auh QS. Volatile sulfide compounds and oral microorganisms on the inner surface of masks in individuals with halitosis during COVID-19 pandemic. Sci Rep. (2023) 13:2487. 10.1038/s41598-023-29080-3 36781937 PMC9924882

[34] Faria SFS Costa FO Pereira AG Cota LOM. Self-perceived and self-reported breath odour and the wearing of face masks during the COVID-19 pandemic. Oral Dis. (2022) 28(Suppl 2):2406–16. 10.1111/odi.13958 34245645 PMC8447418

[35] Tyrrell KL Citron DM Warren YA Nachnani S Goldstein EJ. Anaerobic bacteria cultured from the tongue dorsum of subjects with oral malodor. Anaerobe. (2003) 9:243–6. 10.1016/S1075-9964(03)00109-4 16887710

[36] Au S Baraniya D Dao J Awan SB Alvarez J Sklar S Prolonged mask wearing does not alter the oral microbiome, salivary flow rate or gingival health status—a pilot study. Front Cell Infect Microbiol. (2022) 12:1039811. 10.3389/fcimb.2022.1039811 36439237 PMC9684305

[37] Eribe ERK Olsen I. Leptotrichia species in human infections II. J Oral Microbiol. (2017) 9:1368848. 10.1080/20002297.2017.1368848 29081911 PMC5646626

[38] Ye W Zhang Y He M Zhu C Feng XP. Relationship of tongue coating microbiome on volatile sulfur compounds in healthy and halitosis adults. J Breath Res. (2019) 14:016005. 10.1088/1752-7163/ab47b4 31553956

[39] Socransky SS Haffajee AD Cugini MA Smith C Kent RL Jr. Microbial complexes in subgingival plaque. J Clin Periodontol. (1998) 25:134–44. 10.1111/j.1600-051X.1998.tb02419.x 9495612

[40] Bodet C Chandad F Grenier D. Pathogenic potential of Porphyromonas gingivalis, Treponema denticola and Tannerella forsythia, the red bacterial complex associated with periodontitis. Pathol Biol (Paris). (2007) 55:154–62. 10.1016/j.patbio.2006.07.045 17049750

[41] Chen W Kajiya M Giro G Ouhara K Mackler HE Mawardi H Bacteria-derived hydrogen sulfide promotes IL-8 production from epithelial cells. Biochem Biophys Res Commun. (2010) 391:645–50. 10.1016/j.bbrc.2009.11.113 19932683 PMC2874960

[42] Nakamura S Shioya K Hiraoka BY Suzuki N Hoshino T Fujiwara T Porphyromonas gingivalis hydrogen sulfide enhances methyl mercaptan-induced pathogenicity in mouse abscess formation. Microbiology (Reading). (2018) 164:529–39. 10.1099/mic.0.000640 29488863

[43] Phillips L Chu L Kolodrubetz D. Multiple enzymes can make hydrogen sulfide from cysteine in Treponema denticola. Anaerobe. (2020) 64:102231. 10.1016/j.anaerobe.2020.102231 32603680 PMC7484134

[44] Suzuki N Yoneda M Takeshita T Hirofuji T Hanioka T. Induction and inhibition of oral malodor. Mol Oral Microbiol. (2019) 34:85–96. 10.1111/omi.12259 30927516

[45] Nakano Y Suzuki N Kuwata F. Predicting oral malodour based on the microbiota in saliva samples using a deep learning approach. BMC Oral Health. (2018) 18:128. 10.1186/s12903-018-0591-6 30064419 PMC6069980

[46] Takeshita T Suzuki N Nakano Y Yasui M Yoneda M Shimazaki Y Discrimination of the oral microbiota associated with high hydrogen sulfide and methyl mercaptan production. Sci Rep. (2012) 2:215. 10.1038/srep00215 22355729 PMC3253589

[47] Cui J Cui H Yang M Du S Li J Li Y Tongue coating microbiome as a potential biomarker for gastritis including precancerous cascade. Protein Cell. (2019) 10:496–509. 10.1007/s13238-018-0596-6 30478535 PMC6588651

[48] Ahn J Yang L Paster BJ Ganly I Morris L Pei Z Oral microbiome profiles: 16S rRNA pyrosequencing and microarray assay comparison. PLoS One. (2011) 6:e22788. 10.1371/journal.pone.0022788 21829515 PMC3146496

[49] Jiang B Liang X Chen Y Ma T Liu L Li J Integrating next-generation sequencing and traditional tongue diagnosis to determine tongue coating microbiome. Sci Rep. (2012) 2:936. 10.1038/srep00936 23226834 PMC3515809

[50] Hall MW Singh N Ng KF Lam DK Goldberg MB Tenenbaum HC Inter-personal diversity and temporal dynamics of dental, tongue, and salivary microbiota in the healthy oral cavity. NPJ Biofilms Microbiomes. (2017) 3:2. 10.1038/s41522-016-0011-0 28649403 PMC5445578

[51] Samaranayake LP. Oral candidosis: an old disease in new guises. Dent Update. (1990) 17:36–8. 2198174

[52] Koga C Yoneda M Nakayama K Yokoue S Haraga M Oie T The detection of Candida species in patients with halitosis. Int J Dent. (2014) 2014:857647. 10.1155/2014/857647 25243010 PMC4158284

[53] Awano N Wada M Mori H Nakamori S Takagi H. Identification and functional analysis of Escherichia coli cysteine desulfhydrases. Appl Environ Microbiol. (2005) 71:4149–52. 10.1128/AEM.71.7.4149-4152.2005 16000837 PMC1169034

[54] Tiso M Schechter AN. Nitrate reduction to nitrite, nitric oxide and ammonia by gut bacteria under physiological conditions. PLoS One. (2015) 10:e0119712. 25803049 10.1371/journal.pone.0119712 PMC4372352

[55] Rath S Heidrich B Pieper DH Vital M. Uncovering the trimethylamine-producing bacteria of the human gut microbiota. Microbiome. (2017) 5:54. 10.1186/s40168-017-0271-9 28506279 PMC5433236

[56] Patel J Woolley J. Necrotizing periodontal disease: oral manifestation of COVID-19. Oral Dis. (2021) 27(Suppl 3):768–9. 10.1111/odi.13462 32506662 PMC7301037

[57] Nasiri K Dimitrova A Wrbas KT. Managing halitosis during the SARS-CoV-2 pandemic. J Dent Sci. (2022) 17:1418–9. 10.1016/j.jds.2022.04.020 35530437 PMC9057978

[58] Dziedzic A Wojtyczka R. The impact of coronavirus infectious disease 19 (COVID-19) on oral health. Oral Dis. (2021) 27(Suppl 3):703–6. 10.1111/odi.13359 32304276 PMC7264805

[59] Watanabe H. Observation of the ultrastructure of the tongue coating. Kokubyo Gakkai Zasshi. (2006) 73:26–39. 10.5357/koubyou.73.26 16629468

[60] Xu H Zhong L Deng J Peng J Dan H Zeng X High expression of ACE2 receptor of 2019-nCoV on the epithelial cells of oral mucosa. Int J Oral Sci. (2020) 12:8. 10.1038/s41368-020-0074-x 32094336 PMC7039956

[61] Li Y Cui J Liu Y Chen K Huang L Liu Y. Oral, tongue-coating Microbiota, and metabolic disorders: a novel area of interactive research. Front Cardiovasc Med. (2021) 8:730203. 10.3389/fcvm.2021.730203 34490384 PMC8417575

[62] Liu X Tong X Jie Z Zhu J Tian L Sun Q Sex differences in the oral microbiome, host traits, and their causal relationships. iScience. (2023) 26:105839. 10.1016/j.isci.2022.105839 36660475 PMC9843272

[63] Koliada A Moseiko V Romanenko M Lushchak O Kryzhanovska N Guryanov V Sex differences in the phylum-level human gut microbiota composition. BMC Microbiol. (2021) 21:131. 10.1186/s12866-021-02198-y 33931023 PMC8088078

[64] Tramice A Paris D Manca A Guevara Agudelo FA Petrosino S Siracusa L Analysis of the oral microbiome during hormonal cycle and its alterations in menopausal women: the “AMICA” project. Sci Rep. (2022) 12:22086. 10.1038/s41598-022-26528-w 36543896 PMC9772230

[65] Lim Y Totsika M Morrison M Punyadeera C. Oral microbiome: a new biomarker reservoir for oral and oropharyngeal cancers. Theranostics. (2017) 7:4313–21. 10.7150/thno.21804 29158828 PMC5695015

[66] Cortelli JR Barbosa MD Westphal MA. Halitosis: a review of associated factors and therapeutic approach. Braz Oral Res. (2008) 22(Suppl 1):44–54. 10.1590/S1806-83242008000500007 19838550

[67] Wei W Li J Shen X Lyu J Yan C Tang B Oral Microbiota from periodontitis promote oral squamous cell carcinoma development via γδ T cell activation. mSystems. (2022) 7:e0046922. 36000726 10.1128/msystems.00469-22 PMC9600543

[68] Uppal N Singh P. Oral cancer: breath of death. Br Dent J. (2016) 221:212. 10.1038/sj.bdj.2016.619 27608554

[69] Papapanou PN Sanz M Buduneli N Dietrich T Feres M Fine DH Periodontitis: consensus report of workgroup 2 of the 2017 world workshop on the classification of periodontal and peri-implant diseases and conditions. J Periodontol. (2018) 89(Suppl 1):S173–82. 10.1002/JPER.17-0721 29926951

[70] Kassebaum NJ Smith AGC Bernabé E Fleming TD Reynolds AE Vos T Global, regional, and national prevalence, incidence, and disability-adjusted life years for oral conditions for 195 countries, 1990-2015: a systematic analysis for the global burden of diseases, injuries, and risk factors. J Dent Res. (2017) 96:380–7. 10.1177/0022034517693566 28792274 PMC5912207

[71] Peres MA Macpherson LMD Weyant RJ Daly B Venturelli R Mathur MR Oral diseases: a global public health challenge. Lancet. (2019) 394:249–60. 10.1016/S0140-6736(19)31146-8 31327369

[72] Samaranayake L Matsubara VH. Normal oral Flora and the oral ecosystem. Dent Clin North Am. (2017) 61:199–215. 10.1016/j.cden.2016.11.002 28317562

[73] Pihlstrom BL Michalowicz BS Johnson NW. Periodontal diseases. Lancet. (2005) 366:1809–20. 10.1016/S0140-6736(05)67728-8 16298220

[74] Zhang S Yu N Arce RM. Periodontal inflammation: integrating genes and dysbiosis. Periodontol 2000. (2020) 82:129–42. 10.1111/prd.12267 31850627 PMC6924568

[75] Valm AM Mark Welch JL Rieken CW Hasegawa Y Sogin ML Oldenbourg R Systems-level analysis of microbial community organization through combinatorial labeling and spectral imaging. Proc Natl Acad Sci U S A. (2011) 108:4152–7. 10.1073/pnas.1101134108 21325608 PMC3054005

[76] Mark Welch JL Rossetti BJ Rieken CW Dewhirst FE Borisy GG. Biogeography of a human oral microbiome at the micron scale. Proc Natl Acad Sci U S A. (2016) 113:E791–800. 10.1073/pnas.1522149113 26811460 PMC4760785

[77] Wilbert SA Mark Welch JL Borisy GG. Spatial ecology of the human tongue dorsum microbiome. Cell Rep. (2020) 30:4003–15.e3. 10.1016/j.celrep.2020.02.097 32209464 PMC7179516

[78] Schincaglia GP Hong BY Rosania A Barasz J Thompson A Sobue T Clinical, immune, and microbiome traits of gingivitis and peri-implant mucositis. J Dent Res. (2017) 96:47–55. 10.1177/0022034516668847 28033066

[79] Diaz PI Hoare A Hong BY. Subgingival microbiome shifts and community dynamics in periodontal diseases. J Calif Dent Assoc. (2016) 44:421–35. 10.1080/19424396.2016.12221035 27514154

[80] Hajishengallis G Darveau RP Curtis MA. The keystone-pathogen hypothesis. Nat Rev Microbiol. (2012) 10:717–25. 10.1038/nrmicro2873 22941505 PMC3498498

[81] Coli JM Tonzetich J. Characterization of volatile sulphur compounds production at individual gingival crevicular sites in humans. J Clin Dent. (1992) 3:97–103. 1306680

[82] Yaegaki K Sanada K. Volatile sulfur compounds in mouth air from clinically healthy subjects and patients with periodontal disease. J Periodontal Res. (1992) 27:233–8. 10.1111/j.1600-0765.1992.tb01673.x 1640345

[83] Quirynen M Dadamio J Van Den Velde S De Smit M Dekeyser C Van Tornout M Characteristics of 2000 patients who visited a halitosis clinic. J Clin Periodontol. (2009) 36:970–5. 10.1111/j.1600-051X.2009.01478.x 19811581

[84] Alzoman H. The association between periodontal diseases and halitosis among Saudi patients. Saudi Dent J. (2021) 33:34–8. 10.1016/j.sdentj.2020.02.005 33473240 PMC7801244

[85] Van Gils LM Slot DE Van Der Sluijs E Hennequin-Hoenderdos NL Van Der Weijden FG. Tongue coating in relationship to gender, plaque, gingivitis and tongue cleaning behaviour in systemically healthy young adults. Int J Dent Hyg. (2020) 18:62–72. 10.1111/idh.12416 31309703 PMC7004167

[86] He C Liao Q Fu P Li J Zhao X Zhang Q Microbiological characteristics of different tongue coatings in adults. BMC Microbiol. (2022) 22:214. 10.1186/s12866-022-02626-7 36085010 PMC9461261

[87] Verma D Garg PK Dubey AK. Insights into the human oral microbiome. Arch Microbiol. (2018) 200:525–40. 10.1007/s00203-018-1505-3 29572583

[88] Seerangaiyan K Van Winkelhoff AJ Harmsen HJM Rossen JWA Winkel EG. The tongue microbiome in healthy subjects and patients with intra-oral halitosis. J Breath Res. (2017) 11:036010. 10.1088/1752-7163/aa7c24 28875948

[89] Ren W Xun Z Wang Z Zhang Q Liu X Zheng H Tongue coating and the salivary microbial communities vary in children with halitosis. Sci Rep. (2016) 6:24481. 10.1038/srep24481 27080513 PMC4832241

[90] Su CY Shigeishi H Nishimura R Ohta K Sugiyama M. Detection of oral bacteria on the tongue dorsum using PCR amplification of 16S ribosomal RNA and its association with systemic disease in middle-aged and elderly patients. Biomed Rep. (2019) 10:70–6. 30588306 10.3892/br.2018.1175 PMC6299208

[91] De Boever EH De Uzeda M Loesche WJ. Relationship between volatile sulfur compounds, BANA-hydrolyzing bacteria and gingival health in patients with and without complaints of oral malodor. J Clin Dent. (1994) 4:114–9. 8031479

[92] Kato H Yoshida A Awano S Ansai T Takehara T. Quantitative detection of volatile sulfur compound- producing microorganisms in oral specimens using real-time PCR. Oral Dis. (2005) 11(Suppl 1):67–71. 10.1111/j.1601-0825.2005.01096.x 15752104

[93] Takeuchi H Machigashira M Takeuchi N Nakamura T Noguchi K. The association of periodontopathic Bacteria levels in Saliva and tongue coating with oral malodor in periodontitis patients. Oral Health Prev Dent. (2017) 15:285–91. 28674708 10.3290/j.ohpd.a38529

[94] Ehizele AO Ojehanon PI. Relationship between the concentration of volatile sulphur compound and periodontal disease severity in Nigerian young adults. Niger Med J. (2013) 54:149–52. 10.4103/0300-1652.114564 23901175 PMC3719238

[95] Bokhari SA Khan AA Butt AK Azhar M Hanif M Izhar M Non-surgical periodontal therapy reduces coronary heart disease risk markers: a randomized controlled trial. J Clin Periodontol. (2012) 39:1065–74. 10.1111/j.1600-051X.2012.01942.x 22966824

[96] Ramfjord SP Caffesse RG Morrison EC Hill RW Kerry GJ Appleberry EA 4 Modalities of periodontal treatment compared over 5 years. J Clin Periodontol. (1987) 14:445–52. 10.1111/j.1600-051X.1987.tb02249.x 3308969

[97] Izidoro C Botelho J Machado V Reis AM Proença L Barroso H Non-surgical periodontal treatment impact on subgingival microbiome and intra-oral halitosis. Int J Mol Sci. (2023) 24. 10.3390/ijms24032518 36768839 PMC9916745

[98] Bicak DA. A current approach to halitosis and oral malodor—a mini review. Open Dent J. (2018) 12:322–30. 10.2174/1874210601812010322 29760825 PMC5944123

[99] Ng W Tonzetich J. Effect of hydrogen sulfide and methyl mercaptan on the permeability of oral mucosa. J Dent Res. (1984) 63:994–7. 10.1177/00220345840630071701 6588090

[100] Makino Y Yamaga T Yoshihara A Nohno K Miyazaki H. Association between volatile sulfur compounds and periodontal disease progression in elderly non-smokers. J Periodontol. (2012) 83:635–43. 10.1902/jop.2011.110275 21861638

[101] Tanaka M Yamamoto Y Kuboniwa M Nonaka A Nishida N Maeda K Contribution of periodontal pathogens on tongue dorsa analyzed with real-time PCR to oral malodor. Microbes Infect. (2004) 6:1078–83. 10.1016/j.micinf.2004.05.021 15380777

[102] Musić L Par M Peručić J Badovinac A Plančak D Puhar I. Relationship between halitosis and periodontitis: a pilot study. Acta Stomatol Croat. (2021) 55:198–206. 10.15644/asc55/2/9 34248153 PMC8255038

[103] Stephen AS Dhadwal N Nagala V Gonzales-Marin C Gillam DG Bradshaw DJ Interdental and subgingival microbiota may affect the tongue microbial ecology and oral malodour in health, gingivitis and periodontitis. J Periodontal Res. (2021) 56:1174–84. 10.1111/jre.12931 34486723

[104] Carda-Diéguez M Rosier BT Lloret S Llena C Mira A. The tongue biofilm metatranscriptome identifies metabolic pathways associated with the presence or absence of halitosis. NPJ Biofilms Microbiomes. (2022) 8:100. 10.1038/s41522-022-00364-2 36535943 PMC9763428

[105] Izidoro C Botelho J Machado V Reis AM Proença L Alves R Periodontitis, halitosis and oral-health-related quality of life-A cross-sectional study. J Clin Med. (2021) 10. 10.3390/jcm10194415 34640433 PMC8509422

[106] Bollen CM Beikler T. Halitosis: the multidisciplinary approach. Int J Oral Sci. (2012) 4:55–63. 10.1038/ijos.2012.39 22722640 PMC3412664

